# NMR Characterization of the Interactions Between Glycosaminoglycans and Proteins

**DOI:** 10.3389/fmolb.2021.646808

**Published:** 2021-03-16

**Authors:** Changkai Bu, Lan Jin

**Affiliations:** National Glycoengineering Research Center, Shandong Key Laboratory of Carbohydrate Chemistry and Glycobiology, Shandong University, Qingdao, China

**Keywords:** glycosaminoglycans, proteins, interaction, NMR, conformation

## Abstract

Glycosaminoglycans (GAGs) constitute a considerable fraction of the glycoconjugates found on cellular membranes and in the extracellular matrix of virtually all mammalian tissues. The essential role of GAG-protein interactions in the regulation of physiological processes has been recognized for decades. However, the underlying molecular basis of these interactions has only emerged since 1990s. The binding specificity of GAGs is encoded in their primary structures, but ultimately depends on how their functional groups are presented to a protein in the three-dimensional space. This review focuses on the application of NMR spectroscopy on the characterization of the GAG-protein interactions. Examples of interpretation of the complex mechanism and characterization of structural motifs involved in the GAG-protein interactions are given. Selected families of GAG-binding proteins investigated using NMR are also described.

## Introduction

Glycosaminoglycans (GAGs) are linear acidic heteropolysaccharides that exist in all mammals and are formed by repeating disaccharide units composed of N-acetyl-hexosamine and hexuronic or hexose (Table 1; Vasconcelos and Pomin, 2017). GAGs can have different sulfation patterns with different charge densities and heterogeneous monosaccharide compositions (Uhl et al., 2020). In addition to HA, GAGs are synthesized from the Golgi apparatus in the form of proteoglycans (Sasarman et al., 2016). According to the disaccharide composition and sulfation pattern, GAGs can be divided into several groups, including heparin/heparan sulfate (HS), chondroitin sulfate (CS)/dermatan sulfate (DS), keratan sulfate (KS) and hyaluronic acid (HA) (Pomin and Mulloy, 2018). Heparin/HS is composed of repeating disaccharide units of glucosamine (GlcNAc) and glucuronic acid (GlcA) or iduronic acid (IdoA). The initial substrate is [→4)-β-D-GlcA-(1→4)- α-D-GlcNAc-(1→] n. GlcNAc can be substituted by sulfate groups at the amide, 3 or/and 6 hydroxyl groups, and the persulfation can be written as GlcNS3S6S. GlcA can be converted into IdoA by C5 epimerase, and both can be modified by 2-O-sulfation (written as IdoA2S or GlcA2S). CS consists of repeating disaccharide units of glucuronic acid (GlcA) and galactosamine (GalNAc). The initial substrate is [→4)-β-D-GlcA-(1→3)- β-D-GalNAc-(1→] n. CS can undergo sulfation modification similar to heparin except for N-sulfation. However, due to the difference in glycosidic linkage, 3-O-sulfation in heparin becomes 4-O-sulfation. DS is obtained by converting GlcA in CS by C5-epimerase into IdoA. KS consists of repeating disaccharide units of Gal and GlcNAc, both of which can be 6-O-sulfated (Pomin, 2015). HA is the only GAG that is not modified by sulfation and is not synthesized as proteoglycans. It is composed of repeating disaccharide units of GlcA and GlcNAc. According to the monosaccharide composition and sulfation pattern, GAG disaccharides can have 408 possible compositions (Soares et al., 2017).

**TABLE 1:** Structures and tissue distribution of glycosaminoglycans.

Glycosaminoglycans		Degree of sulfation per disaccharide unit	Molecular weight range	Tissue distribution
Heparin/Heparan sulfate (HS)	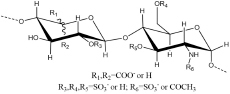	Heparin about 1.8∼2.4	Heparin about 3∼30 kDa	Heparin in liver, lungs and skin;
		HS about 0.8∼1.8	HS about 10∼100 kDa	HS was widely distributed on the cell surface.
Chondroitin sulfate (CS)	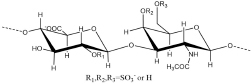	0.1–1.3	5∼50 kDa	cartilage, tendon, aorta, ligament
Dermatan sulfate (DS)	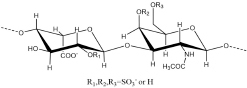	< 1	15∼40 kDa	skin, blood vessels, heart valves
Hyaluronic acid (HA)	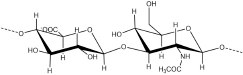	0	4∼12000 kDa	synovial fluid, vitreous humour, ECM of loose connective tissue
Keratan sulfate (KS)	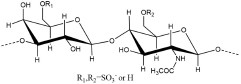	< 1	5∼25 kDa	KS I in cornea; KS II in cartilage aggregated; KS III in brain tissue

As an important component of the extracellular matrix (ECM), GAGs play important roles in the construction of biological systems and the transduction of biological signals (Theocharis et al., 2016). Signal transduction occurs mainly through the interaction between GAGs and proteins, and these interactions are critical to the biological activity of these proteins. GAGs participate in a variety of physiological processes, including binding, activating and fixing a variety of protein ligands, such as growth factors, cytokines, chemokines, lipoproteins, proteases and their inhibitors, and other ECM components (Dyer et al., 2017; Rider and Mulloy, 2017; Crijns et al., 2020). GAGs are also associated with many pathological processes, including degenerative neurological diseases (Alzheimer’s disease), cardiovascular diseases (thrombosis and atherosclerosis) and cancer (Vigetti et al., 2016; Huynh et al., 2019; Morla, 2019). In the invasion of viruses, GAGs also play roles that cannot be ignored (such as in herpes simplex virus and COVID-19) (Liu et al., 2020). The interaction between GAGs and proteins occurs mainly through electrostatic forces. This puts forward requirements for amino acid sequences in proteins and meets some rules, such as the XBBXBX and XBBBXXBX heparin-binding sequences proposed by Cardin, where B is a basic amino acid and X is any amino acid (Cardin and Weintraub, 1989). However, long-term research has found that the interaction between GAGs and proteins is not simply determined by the primary structure sequence. A large number of studies have proven that hydrogen bonds and van der Waals forces sometimes even play roles far exceeding electrostatic forces in the interaction; a proper tertiary structure of the protein is also required (Rudd et al., 2017). This poses more serious and complex problems for studying the interactions between GAGs and proteins.

The interactions between GAGs and proteins are closely related to many factors, including saccharide unit composition, degree of sulfation, sulfation pattern, chain length, monosaccharide ring conformation and glycosidic linkage. The research methods used to characterize the interaction between GAGs and proteins mainly include gel electrophoresis (GE) (Nogueira et al., 2019), affinity chromatography (AC) (Sandoval et al., 2020), surface plasmon resonance (SPR) (Przybylski et al., 2020), biological layer interferometry (BLI) (Xiao et al., 2016), isothermal titration (ITC) (Zsila et al., 2018), microarray methods (Pomin and Wang, 2018b), crystal diffraction methods (X-ray) (Dahms et al., 2015), mass spectrometry (MS) (Yang and Chi, 2017), and nuclear magnetic resonance spectroscopy (NMR) (Kato and Peters, 2017). NMR is an insensitive technique compared with other analytical method for the study of interactions between biomolecules. The amount of sample needs to be in milligrams with high purity. In the study of proteins, NMR can characterize a protein with a molecular weight around 20 KD very well. However, proteins need to be isotope labeled by ^15^N and/or ^13^C when the molecular weight increases and can be studied up to 100 KD. The cross peaks will become broadening and overlapped severely for larger proteins. Even with the above limitations, NMR is still an irreplaceable technique in the characterization of the biomolecule interactions at the atomic level especially in the case of glycosamionoglycans. Both X-ray diffraction and NMR can provide more precise tertiary structure information, and they do not require sample derivatization and will not cause structural damage to the sample during the experiment. Due to the accuracy and refinement of the data, both types of data can be used for model construction. However, X-ray diffraction studies a crystal in solid state and provide only few conformations of the interaction. While, NMR studies a solution under physiological condition and records dynamic conformations during the whole interaction period. Glycosaminoglycans are very hard to obtain a crystal due to their high flexibilities and exchangeable conformations. The solution NMR can not only show the natural state of the complex, but also detect the change of the complex conformation on the ns-ms time scale (Pomin and Wang, 2018a). Compared with the immobilization study of crystal diffraction, solution NMR can also be used for the dynamic study of interactions under physiological conditions.

Nuclear magnetic resonance is widely used to study the conformation of GAGs alone or in complex with proteins (Pomin, 2014), but the information usually obtained indicates that there are multiple GAGs or complex structures in solution. According to NMR data, GAGs present different folds configurations in solution according to their type and environment (Mulloy, 2006), such as the controversial 3-folds and 4-folds coexisting left-handed helix of HA (Gargiulo et al., 2010), which will directly affect the distribution of acidic groups in space. Generally speaking, the conformational changes of GAGs are mainly caused by two factors, one is the ring conformation of monosaccharides, and the second is the flexibility of the glycosidic linkages (Skidmore et al., 2009). The conformation of the IdoA residue in heparin, HS and DS is different from that of the other three monosaccharides (GlcNAc, GalNAc, and GlcA). IdoA exist in the conformational equilibrium, with two chairs (^1^C_4_ and ^4^C_1_) and one shew-boat (^2^S_0_), instead of the fixed conformation ^4^C_1_ adopted in GalNAc, GlcNAc, or GlcA (Pomin, 2014). This gives these three different types of GAGs more flexible and various protein binding activities. This balance is affected by chain length, the degree of sulfation of adjacent monosaccharides, and its own 2-O sulfation (Haasnoot et al., 2020). When interacting with proteins, the conformational balance of IdoA will be tilted, such as binding to fibroblast growth factor-2 (FGF2), fibroblast growth factor-2 receptor (FGF2R), and eosinophil cationic protein (Ecp) (Hricovíni et al., 2002; García-Mayoral et al., 2013). In free state, when the conformational balance ratio is closer to the required binding state, the binding affinity is stronger (Hricovíni et al., 2002). Conversely, when the required conformation of the bound state cannot be achieved, the activity may be completely lost. But even if the protein has a clear tendency to a certain conformation of IdoA, there will generally be a conformational balance. The binding of AT III to heparin requires an absolute ^2^S_0_ conformation, but according to the NMR structure information, there is negligible ^1^C_4_ conformation in the whole binding process (Guerrini et al., 2006). Even though IdoA brings more variable binding conformational selectivity, recent studies have shown that GlcA has a better effect on the overall conformation of GAGs (Whitmore et al., 2020). In order to adapt to the ECM environment, the angle of the glycosidic linkages is allowed to change to a certain extent. The angle of the glycosidic linkages is affected by temperature, and the increase in temperature will result in a transition to the higher energy state (Hughes et al., 2017). When interacting with proteins, the glycosidic linkages can adopt proper orientations to meet the structural requirements during binding to proteins, and even cause the kinking of the GAGs polymer chain, thereby further enhancing the binding affinity (Hricovini, 2004). Compared with the obvious conformational equilibrium of IdoA, sometimes GAGs have α/β isomeric equilibrium at the reducing end (Silipo et al., 2008) and rapid intramolecular hydrogen bond exchange (Almond et al., 1998). Due to the flexibility of GAGs, there may be multiple interaction modes at the same binding domain in the GAG-protein interaction process (Tjong et al., 2007). In the interaction between GAGs and proteins, the structure of the proteins is normally changed or stabilized. The weak interaction between GAGs and proteins undergoes on the ns-ms time scale, so the conformation of the protein in the system will change over time. Due to the structural heterogeneity and conformational flexibility of GAGs or the dynamic changes of the complex, it is also very difficult to construct a model of complexes in solution (Almond, 2018).

Solution NMR can provide information about conformational changes and kinetic data during interactions between proteins and GAGs (Pomin and Wang, 2018a). NMR can also reveal the effects of different temperatures, pH values, salt concentrations, and ligand concentrations on the binding activity. There are three main goals in using NMR to study GAG-protein interactions: the first is to detect the amino acids involved in binding from the perspective of proteins, the second is to analyze the saccharide and its groups involved in binding from the perspective of GAGs, and the third is to observe the conformational changes and kinetic information during binding from the perspective of the interaction. To achieve these three goals, three technologies, chemical shift perturbation (CSP), saturation transfer difference (STD), and exchange-transferred nuclear Overhauser effect (trNOE), are initially used (Vignovich and Pomin, 2020), while other technologies, such as saturation transfer double difference (STDD) (Ledwitch et al., 2016), paramagnetic relaxation enhancement (PRE) (Orton et al., 2016), pseudocontact shifts (PCS) (Srb et al., 2019), and exchange-transferred rotating-frame Overhauser effect (ROE), have been developed to compensate for the shortcomings of the former. The latest pulse sequences have been developed to provide a more detailed and accurate description of the binding process, such as the gradient spectroscopic observation of water ligands (waterLOGSY) (Huang and Leung, 2019) and heteronuclear in-phase single quantum coherence experiment (HISQC) (Sepuru et al., 2018a). In addition, solid-state NMR has also been applied to study interactions involving ligands with low solubility (Malmos et al., 2016; Stewart et al., 2016). These techniques are based on four types of data: nuclear Overhauser effect (NOE), scalar coupling (*J*), residual dipole coupling (RDC) and chemical shift anisotropy (CSA). The purpose of this paper is to introduce some important findings of the application of NMR to the study of the interactions between GAGs and proteins (Table 2) and the review is classified according to the type of GAGs.

**TABLE 2:** GAG binding proteins.

Type of protein	Name of protein	Type of GAG	participating binding residues and Secondary structure	Affinity (K_d_)	References
Chemokine	CCL5	Heparin	40S loop (R^44^KNR^47^), α helix (K^55^, K^56^)	18 μM	Wang et al., 2011
		CS	40S loop(R^44^KNR^47^), N loop (R^17^, L^19^, I^15^)	0.25 μM	Deshauer et al., 2015
	CXCL1	Heparin/HS	N terminus(R^8^), N-loop (H^19^, K^21^),40S turn (K^45^, R^48^), β3-strand (R^49^), C-helix (K^60^, K^61^, K^65^)	50 μM	Sepuru and Rajarathnam, 2016; Sepuru et al., 2018b; Sepuru and Rajarathnam, 2019
		CS/DS	N terminus(R^8^), N-loop (H^19^, K^21^), 40S turn (R^48^),	4 μM	Sepuru and Rajarathnam, 2019
	CXCL2	Heparin	N-loop (R^17^, K^21^), 40S turn (K^45^), C-helix (K^61^, K^65^, K^69^)	25 μM	Sepuru et al., 2018b
	CXCL5	Heparin/HS	N-loop (H^23^, K^25^), 40S turn (K^49^), β3 strand (K^52^), C-helix (Lys^64^, Lys^65^, Lys^69^, Lys^76^)	30 μM	Sepuru et al., 2016; Sepuru and Rajarathnam, 2019
		CS/DS	N-loop (H^23^, K^25^), 40S turn (K^49^), β3 strand (K^52^)	3 μM	Sepuru and Rajarathnam, 2019
	CXCL7	Heparin	N-loop (H^15^, K^17^), β3-strand(R^44^), C-helix (R^54^, K^57^, K^61^)	—	Brown et al., 2017
	CXCL8	Heparin	N-loop (K^15^, H^18^, K^20^, K^23^), C -helix (R^60^, K^64^, R^68^), β3-strand (R^47^), 50S loop (K^54^)	μM	Joseph et al., 2015
	CXCL11	Heparin	C- helix (K^57^SKQAR^62^)	—	Severin et al., 2010
	CXCL12	Heparin	C-helix (R^12^, A^40^), 20S loop (K^24^), 40S loop (N^46^)	μM	Laguri et al., 2011
	CXCL13	HS	C-helix (K^60^, R^64^, R^67^, H^68^), C-loop (K^84^, R^85^, R^86^)	19 nM	Monneau et al., 2017
	CXCL14	Heparin	10S loop(I^12^), β2-strand (I^36^, T^37^), 40S loop(K^54^), C -helix (R^72^),	—	Penk et al., 2019
		CS/DS	10S loop(I^12^), 40S loop(K^54^), C -helix (R^72^)	—	Penk et al., 2019
Growth factor	FGF1	Heparin	β1–β2 loop (N^18^), β8–β9 loop (N^92^), β10–β11 loop (K^113^), β11 strand (K^118^), β11–β12 loop (Q^127^, K^128^)	nM	Ogura et al., 1999
	FGF2	Heparin	β1 strand (K^27^), β1–β2 loop (N^28^), β8–β9 loop (N^102^), β10–β11 loop (R^121^), β11 strand (K^126^), β11–β12 loop (Q^135^, K^136^)	nM	Faham et al., 1996
	FGF7	Heparin	β3 strand (R^18^), 40s loop(N^92^), β10(N^114^), 110s loop(Q^115^), 120s loop (V^120^, K^124^, Q^129^, K^130^, T^131^)	—	Ye et al., 2001
Serpin	AT III	Heparin	N-terminal end (K^11^, R^13^), A helix (R^46^, R^47^), D helix (K^114^, K^125^, R^129^, R^132^, K^133^, K^136^)	20 nM	Olson et al., 2002
Type II cytokines	IL-10	Heparin/CS/DS	D helix (K^99^, R^102^, R^104^, R^106^), 110S loop (R^107^, K^117^, K^119^)	0.41 mM	Künze et al., 2016
	IFNγ	Heparin	C-terminal end (D1:K^125^TGKRKR^131^, D2:R^137^GRR^140^)	1.63 nM	Saesen et al., 2013
	Roundabout 1	HS	80s loop (K81), 130s loop (V^133^, H^134^, G^135^, R^136^, K^137^), βA strand (I^167^, R^169^)	—	Gao et al., 2016
Cytokine	Pleiotrophin	CS	C-terminal TSR domain β-sheet (K^60^, K^61^, K^69^, K^91^, K^92^, K^84^, K^86^, K^107^)	90 μM	Ryan et al., 2016
Link protein	CD44	HA	β1 strand (K^38^), 40s loop (R^41^, Y^42^), 70s loop (R^78^, Y^79^), 90s loop (N^100^, N^101^), 150s loop (R^150^), β9 strand (R^154^), 160s loop(R^162^)	μM	Banerji et al., 2007
	TSG-6	HA	10s loop (K^11^, Y^12^), 40s loop (H^45^, C^47^), β3 strand (A^49^), β3 strand (Y^59^), 60s loop (V^62^, K^63^), 80s loop (Y^78^, R^81^)	μM	Higman et al., 2014
Viral pathogen	viral CCL2	Heparin	10s loop(R^18^), 40s loop (K^45^, R^46^, R^48^)	113 mM	Zhao and LiWang, 2010
Defensins	Human β-defensin 2	Heparin/DS	20s loop(R^22^RYK^25^), β3 strand (K^39^), 40s loop(K^40^)	5 mM	Seo et al., 2010
RNase A	Eosinophil cationic protein	Heparin	α1 helix (R^7^, Q^14^, H^15^), β1 strand (Q^40^), loop4(H^64^), β6 strand(H^128^)	15 μM	García-Mayoral et al., 2013

## Heparin/Heparan Sulfate

Heparin is the most negatively charged polymer found in nature, and it is also the most studied in the GAG family (Conrad, 1997). One way to distinguish between heparin and HS is based on whether the mature body is still connected to the core protein. HS will be secreted out of the cell in the form of glycoproteins, most of which are fixed on the cell membrane to mediate many intercellular signaling pathways. Heparin is cleaved by β-endoglucuronidase and is combined with alkaline protease in the form of oligosaccharide chains to be stored in secretory granules (Oduah et al., 2016). The binding of heparin to protein mostly relies on its own high electronegativity and the positively charged domains in the protein. Hydrogen bonds and van der Waals forces also play important roles in the binding process. Moreover, the binding of heparin and protein is sometimes ion-dependent. For example, the binding of Langerin and heparin is mainly Ca^2 +^ -dependent, although there are additional non-Ca^2 +^ -dependent binding sites (Muñoz-García et al., 2015; Hanske et al., 2017; José García-Jiménez et al., 2019). HS can be divided into a high-sulfation domain (NS domain) and a low-sulfation domain (NA domain). Heparin essentially contains all possible sulfation modification structures of the NS domain due to the degree of high sulfation. Most of the biological functions of HS are concentrated in the NS domain, although the NA domain is more flexible and more suitable for bending. Due to the early large-scale clinical application of heparin, it was relatively easy to obtain. Early research mainly used heparin as a substitute for HS to carry out functional and structural studies. In approximately the past thirty years, the study of the interaction between heparin and various proteins has become a hot spot, and the gradual maturity of chemical enzyme synthesis has given this field new vitality. Heparin can induce the oligomerization or heteromerization of proteins, which can prevent proteins from being hydrolyzed by protein-degrading enzymes and increase or decrease the possibility of their binding to receptors.

**FIGURE 1:**
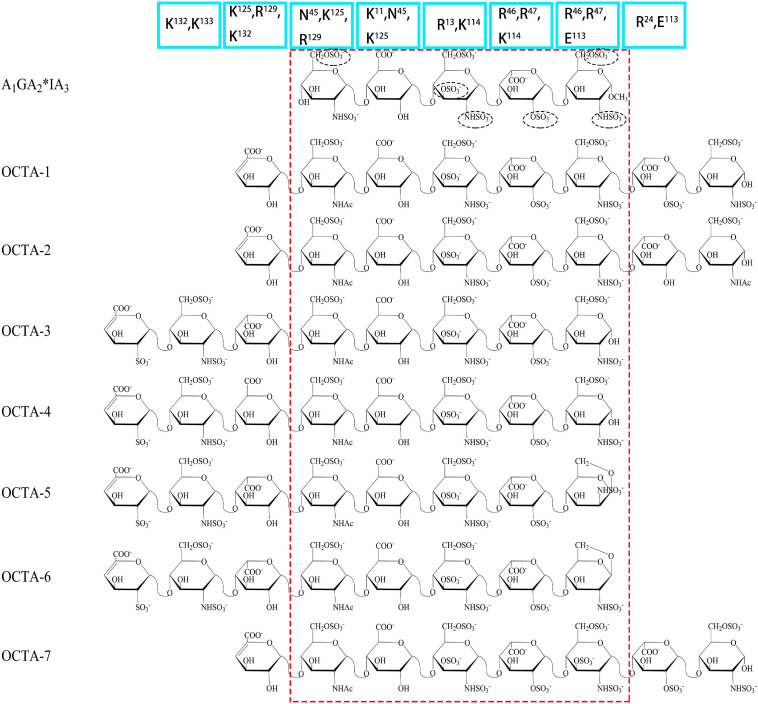
Structures of AT III binding pentasaccharides (red circle) and their extended octasccharides. Sulfate groups in black dashed circles in the pentasaccharide, A_1_GA_2_*IA_3_, are essential for the binding to AT III. Bule squares showed the important amino acids in AT III contributing to the binding to heparin.

**FIGURE 2:**
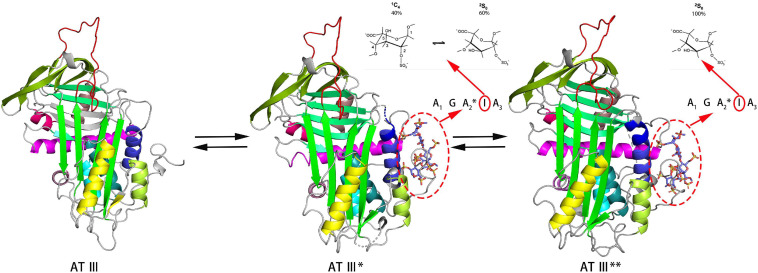
Process of heparin binding to AT III. The binding of heparin with AT III is a reversible process. This process involves native unactivated (AT III, PDB code 1E05), intermediate-activated (AT III*, PDB code 1NQ9) and fully activated (AT III**, 1E03) states. During the binding process, IdoA transforms from conformational equilibrium to a complete ^2^S_0_ conformation (Jimenez-Barbero and Peters, 2003). The models of the three states are derived from X-ray. The reactive center loop (RCL) (red), sheet A (green), and helix D (gray blue) and the helix extension (dark blue) are highlighted in each state.

Antithrombin III (AT III) is an absolutely conserved serine protease with two different glycosylation forms (α, β), consisting of three β-sheets (A-C) and nine α-helices (A-I) (Rezaie and Giri, 2020). Heparin is a cofactor of the antithrombin-mediated coagulation cascade, and the interaction between them directly affects the activities of factors IXa, Xa and IIa (Gray et al., 2012). Choay, J used chemical enzymatic synthesis of various heparin-related oligosaccharides to determine that the minimum specific sequence required for binding to AT III was the pentasaccharide A_1_GA_2_^∗^IA_3_ (Figure 1), which is also the only specific recognition sequence for heparin and protein binding found thus far (Thunberg et al., 1982; Choay et al., 1983). Although the specific pentasaccharide can meet the requirement of binding to AT III, it can only inhibit the activity of Xa. Inhibiting thrombin activity requires a heparin chain containing more than 16 saccharides, which can form a ternary complex with antithrombin and thrombin (Lane et al., 1984). The interaction between heparin and AT III was described as a three-state, two-step kinetic process (Figure 2; Olson et al., 1981), which assumed that AT III was in a balance of ‘native unactivated,’ ‘intermediate-activated’ and ‘fully activated’ states under physiological conditions (Roth et al., 2015). First, A_1_GA_2_^∗^ was driven by K^125^ and K^114^ to combine with the C- terminus of helix D in “native unactivated” AT III, and the reducing end faced the N-terminus (Desai et al., 1998). Then, accompanied by conformational changes in AT III (helix D extension, reactive center loop exposure, and closure of sheet A) and heparin (IdoA from equilibrium conformation between^ 1^C_4_ and ^2^S_0_ to complete ^2^S_0_), each unit in the pentasaccharide was further combined with AT III (van Boeckel et al., 1994). The combined complex can interact with the target protease or enzymatically decompose, and heparin is dissociated accordingly. In the electrostatic binding of heparin and AT III, several sulfate groups of heparin-specific pentasaccharide (N-SO_3_ for A_2_^∗^ and A_3_, 6-O-SO_3_ for A_1_, and 3-O-SO_3_ for A_2_^∗^) and carboxyl groups were irreplaceable (Olson et al., 2002).

Further research using NMR focused on the specific role of each monosaccharide in the binding of heparin to AT III and the effect of extended pentasaccharide on the binding. The ratio of the ^2^S_0_ conformation in IdoA in the A_1_GA_2_^∗^IA_3_ sequence was 20% higher than that in the general heparin sequence (Ferro et al., 1987). In the three different chemically synthesized heparin pentasaccharides, the pentasaccharide had anticoagulant activity only when IdoA was in ^2^S_0_ (Das et al., 2001). Therefore, the proportion of ^2^S_0_ of IdoA in the heparin pentasaccharide sequence was one of the factors affecting the binding rate, which was affected by the degree of sulfation of glucosamine on both sides and its own 2-O-SO_3_ (Haasnoot et al., 2020). Although the absence of 2-O-SO_3_ in IdoA had no significant effect on the binding conformation, it resulted in a decrease in the proportion of free state ^2^S_0_ and a two-fold decrease in affinity (Stancanelli et al., 2018). At the same time, the flexibility of IdoA provided unlimited possibilities for the binding of heparin to protein. A recent study used IdoA2S instead of GlcA in the AT III binding sequence (Elli et al., 2020). The results showed that IdoA2S, which replaced GlcA, was in a pure ^1^C_4_ conformation when bound, and the affinity was tripled, which provided a basis for the application of bovine heparin. The unique structure of bovine heparin also provided unique ideas for the study of the specific mechanism of anticoagulation between heparin and AT III (Naggi et al., 2016). The 3-O-SO_3_ and 6-O-SO_3_ also had significant effects on the conformational balance of IdoA (Muñoz-García et al., 2012; Guerrini et al., 2013). The contribution of A_2_^∗^’s 3-O-SO_3_ to binding was in not only the conformation of heparin but also the formation of ‘intermediate-activated’ AT III (Lindahl et al., 1980; Casu et al., 1981). Octa-7 (Figure 1), an octasaccharide with extended reducing end, showed that adding an extra 3-O-SO_3_ to the A_3_ would increase the ratio of ^2^S_0_ in I by approximately 15%. The additional 3-O-SO_3_ formed new ionic bonds with R^46^ and R^47^. The extended disaccharide also had a certain contribution to the binding (by interacting with E^113^ and R^24^), and the binding force of otcasccharide and AT III was 40% higher than that of the specific pentasaccharide sequence and AT III. In the binding state, I and extended nonreducing end IdoA2S was completely in ^2^S_0_. In a similar structure (OCTA-1), due to the lack of 3-O-SO_3_ in the reducing end of A_3_, the extended IdoA2S was completely in ^1^C_4_ when bound, resulting in a substantial decrease in affinity (Guerrini et al., 2013). When extended reducing end IdoA2S’s 2-O-SO_3_ was removed (OCTA-2), the affinity increased slightly (Guerrini et al., 2008). In addition, there was little interaction between the reducing end extended disaccharide and AT III. In the other two octasaccharides with GlcA or IdoA as the extended nonreducing end (OCTA-3, OCTA-4), there was a significant polarization of affinity. The affinity of octasaccharide with GlcA as the nonreducing end was one order of magnitude higher than that with IdoA, which was in pure ^2^S_0_. In recent years, the appearance of low-molecular-weight heparin has become a research hotspot due to its unique fragments produced by cleavage or hydrolysis on anticoagulation. In Guerrini’s study, the affinity of two octasaccharides (OCTA-5, OCTA-6) containing specific pentasaccharide sequences derived from enoxaparin in binding with AT III decreased by 60-fold compared with the hexasaccharide with a complete pentasaccharide sequence. Because of the special pentasaccharide unit, the binding of the reducing end became weaker (Guerrini et al., 2010). The interaction difference of the octasaccharides with AT III showed that the substitution of different groups on heparin not only affected the binding strength with AT III but also changed the conformation during binding.

Heparin plays a key role in the regional aggregation and oligomerization of fibroblast growth factor (FGF), protecting it from denaturation and degradation and inducing its binding to the receptor (FGFR) (Korsensky and Ron, 2016). FGF is a growth factor family with 23 members, and its structure is highly related (12 β strands form the classic β-trefoil structure) (Li et al., 2016). The receptor proteins of FGF include four categories (FGFR1-4), which are composed of three immunoglobulin (Ig)-like domains, which can be subdivided into seven categories according to the difference in Ig3 (Cheng et al., 2017). FGFR Ig2 is a key site for the binding of FGF and FGFR mediated by heparin (Kan et al., 1993). In the study of the effect of FGF and heparin, acidic fibroblast growth factor (aFGF, FGF1) and basic fibroblast growth factor (bFGF, FGF2) were the most classic models (Schlessinger et al., 2000). Studies have shown that the binding of heparin to FGF does not change the FGF conformation, and the binding domain is mainly located at the β1-2 and β10-11 strands (Canales-Mayordomo et al., 2006). Although there is clear evidence in the study of Crystallography, in the free state, 116-120 (131-136) of FGF1 (FGF2) constitute βXI structure (Zhu et al., 1991). However, Moy’s NMR study on the structure of FGF2 in solution showed that there was no evidence to prove the existence of βXI (Moy et al., 1995). It is speculated that this is the structural change caused by the combination with HSPG, and this change is very important for the combination. This was confirmed in the subsequent NMR structural study of FGF1, Ogura pointed out that in the binding state, the 116-120 sequence has an obvious tendency of β-chain structure (Ogura et al., 1999). In addition, K^125^ in FGF2 and K^118^ in FGF1 had high affinity in binding with heparin. Therefore, the β11 chain was considered to be the key structure for the binding of FGF to heparin. In the combination of FGF2 and heparin, 2-O-SO_3_ and N-SO_3_ were necessary (Yu et al., 2014), and additional 6-O-SO_3_ was required for FGF1 (Guerrini et al., 2002). However, in the study using 48 kinds of heparin disaccharides to bind FGF1, 3-O-SO_3_ provided a stronger binding ability, and further C6 sulfation seemed to have a negative effect on the binding (Hu et al., 2012). In the study of the binding of heparin to FGF, ^1^C_4_ might have been the more favorable conformation (Canales et al., 2005; Guglieri et al., 2008). Interestingly, a recent study showed that specific AT-binding sequences can bind to FGFR2 Ig2 as a high-affinity complex, and IdoA remained in a high proportion of ^2^S_0_ (Nieto et al., 2011). Some experiments have shown that the combination of FGF and heparin seem to require a certain regular sequence of monosaccharide units or a special sulfation pattern (Ojeda et al., 2002). The mirror image of the carbohydrate structure also caused a significant reduction or loss of activity (Muñoz-García et al., 2013). For FGF1, only a single 6-sulfated tetrasaccharide was needed to induce its dimerization (Hricovíni et al., 2002). However, for FGF2 to be fully activated, heparin fragments of approximately decasaccharide might be required (Moy et al., 1997), although there was also evidence that tetrasaccharides could induce FGF2 dimerization (Guglieri et al., 2008). Heparin can induce FGF dimerization, but whether it is a critical step is controversial. Some NMR data showed that heparin, which formed a high-affinity complex with FGF, did not induce the dimerization of FGF but still had high activity (Canales et al., 2006).

**FIGURE 3:**
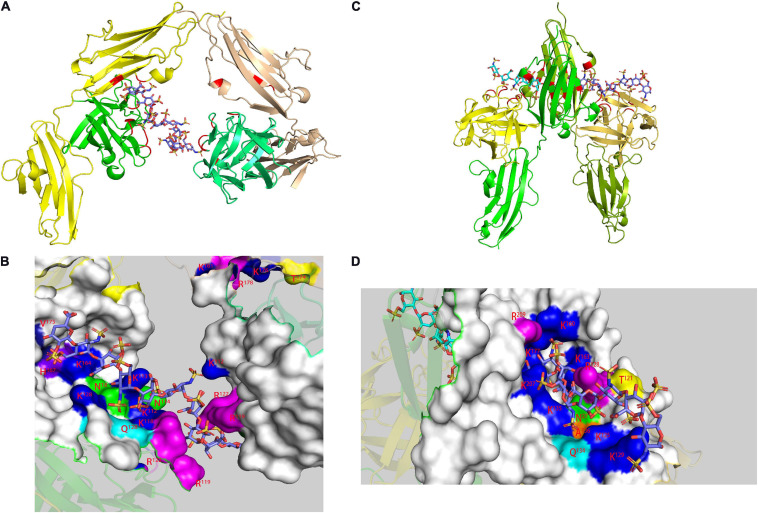
Model of FGF-FGFR-heparin complex obtained by X-ray. FGF1-FGFR2-heparin decasaccharide **(A)** (PDB code 1E0O) and its amplified figure **(B)**, FGF2-FGFR1-heparin decasaccharide **(C)** (PDB code 1FQ9) and its amplified figure **(D)**. In the carton models, the heparin binding domains are shown in red. In the amplified figures, different kinds of heparin binding domains are shown in different colors according to the amino acid residues.

In the study of the FGF-FGFR-heparin binding model (Figure 3), the crystal study gave two hypotheses: a 2:2:1 trans-binding model and a 2:2:2 cis-binding model (Pellegrini, 2001). NMR research in recent years has explained the formation process of the 2:2:2 model. Nieto used FGF1 and FGFR2 Ig2 and two heparin oligosaccharides to study the mechanism (Nieto et al., 2013). In the activity experiment, FGF1 and FGF2 had different requirements for heparin. In deheparinized cells, FGF2 activity was completely lost. However, after pretreatment of the cells with heparin, the activity recovered. FGF1 requires the presence of an additional heparin-like stabilizer myo-inositol hexasulfate (MIHS). It is speculated that the role of heparin in FGF1 was not limited to mediating the binding of FGF and FGFR. There was a second binding site in the FGF-FGFR complex, which was a clear cis-dimer binding model mark. Subsequent speculation suggested that the signaling pathway should be regarded as follows: FGFR dimerization was initially induced by GAGs, and then FGF and the ternary complex formed a higher-order aggregate and activated the subsequent enzyme cascade. Schieborr investigated the interactions among FGF1/FGF2, FGFR4 Ig2, and three different heparin polysaccharides (Saxena et al., 2010). The experimental results showed that the hexasaccharide could meet all the binding site requirements for inducing FGF dimerization, but the stability of the resulting complex was extremely poor. STD experiments showed that the combination of octasaccharide and FGF2 had a positive synergistic effect, but due to the lack of heparin structure data, the exact mechanism needs further experimental verification. Heparin was proven to have an extremely low dimerization ability for inducing FGFR4 Ig2, which was clear proof of the trans-dimer model in the description by Pomin (2016). However, the NMR data suggested there was a secondary binding site in the FGF-FGF Ig2 complex, which was again a clear cis-dimer binding model. Schieborr proposed that hexasaccharides and octasaccharide could mediate FGF2 signaling pathways under different mechanisms, and the positive synergistic effect of octasaccharide was due to the different residues involved in the binding. However, while there should theoretically be an FGF/FGFR/heparin 4:2:2 complex in the pathway, there were no data to support its existence. The existence of the FGF/FGFR/heparin 2:2:1 model was clearly supported by Brown’s ITC data, but no NMR evidence was obtained (Brown et al., 2013).

CXCL12 has six different splicing variants (CXCL12α-φ) in humans and is the only CXC chemokine with differential gene splicing (Janssens et al., 2017b). The complex of CXCL12 and the receptor CXCR4 mediates many physiological functions, including physiological processes such as hematopoiesis, embryonic development, vascular repair, and inflammation (Murphy and Heusinkveld, 2018). CD26, a leukocyte-activating antigen, can be cleaved CXCL12 between the N-terminal P^2^ and V^3^ residues (Janssens et al., 2017a). The cleaved product has a reduced affinity for CXCR4 and cannot activate it any more. Research on the binding domain of CXCL12 and heparin/HS can be traced back to 1999. The K^24^HLK^27^ base sequence in the β1-strand of the β-sheet, conforming to the BBXB rule, was verified in a mutation experiment (Amara et al., 1999). Sadir believed that R^41^ and R^43^ in the β2 strand were additional binding sites, in addition to K^1^ at the N-terminus as a potential binding site (Sadir et al., 2001). The binding between heparin/HS and K^1^ in CXCL12 was believed to protect CXCL12 from being cleaved by CD26 (Sadir et al., 2004). Murphy first used X-ray crystallography to study the interaction between CXCL12 and heparin/HS and proposed two binding domains in CXCL12: one at the interface of the dimer and the other in the N-loop region and the N-terminal helix similar to the binding domain in CXCL8 (Murphy et al., 2007). Using ^13^C-labeled octasaccharides in the NMR experiment, Laguri determined that the heparin-binding sequence was related to the GlcN-3, GlcA-4, and GlcN-5 units of the octasaccharides (Laguri et al., 2011). N-sulfation and 6-O-sulfation are essential for binding. The nonreducing end monosaccharide and reducing end disaccharide of the octasaccharide formed additional contact with the N-terminus of CXCL12 (R^8^ and R^12^ are the most prominent), and a consistent molecular binding model was constructed. However, Ziarek proposed a controversial molecular model (Ziarek et al., 2013). He believed that heparin and two CXCL12 molecules should drive the formation of the polymer in an almost orthogonal conformation, instead of the previously proposed interface of two CXCL12 molecules (composed of a β1 strand and the N-terminus). The data indicated that the binding site in CXCL12 should be on the six-strand of the β-sheet, while the N-terminus was not involved. The main residues involved in binding included K^20^, K^24^, K^27^, K^41^, K^43^ and R^47^, while A^8^ and A^12^ provided additional binding. It was proposed that the reason why heparin protected CXCL12 from CD26 cleavage was not the preemptive combination but the coverage of K1 caused by dimerization. Panitz’s study proved that the interaction affinity between heparin and CXCL12 was much higher than that of other GAGs, and the degree of sulfation was not the only factor influencing the binding (Panitz et al., 2016). The binding sites in CXCL12 with other GAGs were similar to heparin, with the exception of a second binding site for CS compared to heparin (R^20^, A^21^, N^30^, K^64^).

Type II cytokines have six secondary structure elements (A-F) to form an α-helical structure, of which A, C, D, and F adopt the classic four-helix topology, while B and E exist as the connecting structure (Pestka et al., 2004). Interleukin-10 (IL-10), interferon-γ (IFNγ) and interleukin-26 (IL-26) are the three proteins in this family that exist in the form of dimers. Although IL-10 and IFN-γ had the same protein folding mode, their binding with heparin split into two completely different manners. STD data indicated that when IL-10 bound to heparin, the degree of sulfation rather than the site had a greater impact on the binding (Künze et al., 2014), although the effect of 6-O-SO_3_ on affinity was 2-3 times greater than the effects of N-SO_3_ and 2-O-SO_3_. Data showed that there was a hydrogen bond or strong van der Waals force between IL-10 and the methyl group in the N-acetyl residue of the saccharides. As the heparin chain length increases, the affinity increases. When the chain length reached eight sugars, the affinity suddenly increased. It was calculated using STD data that when IL-10 bound to a heparin oligosaccharide with more than eight sugars, the Hill coefficient was approximately 2. This indicated that heparin and each monomer of the IL-10 dimer were bound, and the binding was synergistically positive. It was speculated that the binding site in IL-10 was located at the C-terminus of the D helix and the basic amino acid cluster L^101^RLRLRRCHRF^111^ of the adjacent DE loop. This heparin-binding domain existed in both monomers, which also supported the positive synergistic combination of octasaccharide and IL-10. NOE data showed that the conformation of a tetrasaccharide in the binding center did not change much. Further PCS data confirmed that the binding domain of IL-10 with heparin was in the 101-111 basic amino acid cluster (Gehrcke and Pisabarro, 2015). This domain is absolutely conserved in IL-10 from various sources, and it is also located in the binding domain of IL-10R2 and IL-10. The reason why GAG had an inhibitory effect on IL-10 might be due to the low-affinity IL-10R2 competing with heparin for binding.

Unlike IL-10, the binding domain of IFN-γ with heparin was located at the C-terminus. IFN-γ had four clusters of enriched basic amino acids, but only two C-terminal domains, K^125^-R^131^ (D1) and R^137^-R^140^ (D2), interacted with heparin (Vanhaverbeke et al., 2004). NOE data showed that the interaction between the protein and heparin had no effect on the conformation of the protein, and only the electrostatic force contributed to the binding without any other interaction force. The increase in sugar chain length increased not only the affinity between heparin and IFNγ but also the bending degree of the whole sugar chain. The binding of IFNγ to heparin protected the D1 domain from protease hydrolysis, and D1 acts as the main binding domain to heparin. ITC experiments have shown that D2 is not necessary for the binding of IFNγ to heparin, but removing D2 will increase the binding of IFNγ to heparin (Döbeli et al., 1988). Further studies have shown that the combination of D1 with heparin was mainly a thermodynamic process, while the combination of D2 with heparin was a kinetic process (Saesen et al., 2013). The main function of D2 was to strengthen the binding of IFNγ with heparin. The binding of the C-terminus of IFNγ to heparin is a two-step process. First, D1 bound to heparin, and the binding site was oriented. Then, D2 combined with heparin to strengthen the binding. The binding of IFNγ to its receptor includes two domains, one of which is the C-terminus. Therefore, HSPG on the cell surface competed with the IFNγ receptor for binding; and the addition of exogenous heparin could also reduce the IFNγ concentration on the cell surface. The inhibitory effect of heparin on the activity of certain proteins might be due to its competition with the protein receptor for binding, which led to the decreased or even disappearance of the binding affinity between the receptor and the protein. IL-10 inhibits the activity of IFNγ, so its mechanism might be more complicated. Studying the interaction between GAGs and proteins of a specific sequence may help to develop a more thorough understanding of the mechanism.

## Chondroitin Sulfate

According to the type of uronic acid and sulfation, common CS can be divided into five categories: nonsulfated chondroitin sulfate (CS-O), 4-O-sulfated chondroitin sulfate (CS-A), 6-O-sulfated chondroitin sulfate (CS-C), 2, 4-O-disulfated chondroitin sulfate (CS-D), and 4,6-O-disulfated chondroitin sulfate (CS-E) (Yang et al., 2020). CS-B (DS) has all of the sulfation modification types of the above five types of CS, but its uronic acid is epimerized into IdoA. Oversulfated chondroitin sulfate (OSCS) was sulfated at all sites that could be sulfated, and it was one of the culprits that triggered the “heparin crisis” in 2008 (Zhu et al., 2019). There is a special kind of 3-O-sulfated chondroitin sulfate (CS-K) in marine organisms that has a high affinity for growth factors (Palhares et al., 2019).

**FIGURE 4:**
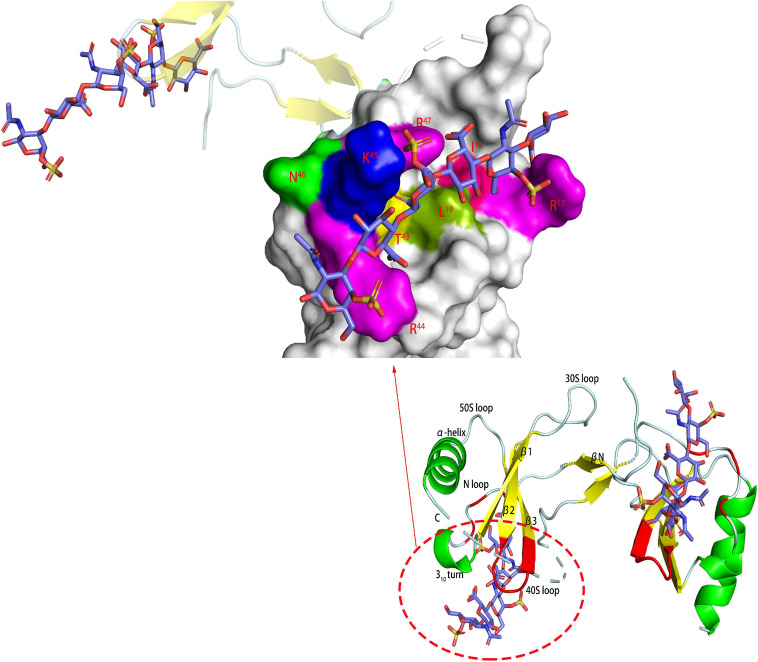
Complex of CCL5 dimer and CS466. In the carton models, the chondroitin sulfate binding domains are shown in red. In the amplified figures, different kinds of chondroitin sulfate binding domains are shown in different colors according to the amino acid residues.

In the interaction with chemokines, the main function of GAG was to locally aggregate chemokines to increase their binding to G-coupled protein receptors and to form a concentration gradient required for the migration of leukocytes, among which HS was dominant (Rajarathnam et al., 2018). However, CS also played an important role in the interaction with certain chemokines, such as the chemokine CCL5 (regulated upon activation of normal T cell expressed and secreted factor, RANTES). CS plays an important role in a variety of biological pathways mediated by CCL5, such as inducing T cell apoptosis and monocyte blockade. Deshauer studied the interaction between two CS hexasaccharides and CCL5 and used TEMPO to label CS for PRE experiments to study the binding sites in depth (Deshauer et al., 2015). In the titration of CCL5 with CS444 (GlcA-GalNAc4S- GlcA-GalNAc4S- GlcA-GalNAc4S), there were obvious chemical shift changes in the 40S loop, the N-terminus and the N loop (Figure 4). At a ratio of 1:1, the chemical shift had no significant change. When CS644 (GlcA-GalNAc6S- GlcA-GalNAc4S- GlcA-GalNAc4S) is used for titration, there are only small chemical shift disturbances at these three binding sites. However, when the ratio of CS644:CCL5 was more than 1:1, R^17^ and L^19^ in the N loop showed obvious chemical shift disturbances. In the PRE experiment, CS444 data showed that its reducing end was close to the 40S loop BBXB sequences. However, CS644 had additional chemical shift changes at Y^3^, A^16^, and R^21^, indicating that CS644 was also close to the 20S loop, N-loop and N-terminus, which suggested that the combination of CS644 and CCL5 was more heterogeneous. It can be seen that the type of GAG, the degree of sulfation and the ring conformation had a huge influence on the binding conformation between GAG and protein, which was also reflected in Pichert’s CXCL8 and CS hexasaccharide interaction study (Pichert et al., 2012).

Midkine (MK) and pleiotropic protein (PTN) form the MK/PTN cytokine family, which is a heparin-binding nerve growth factor. They are highly similar in structure and share more than 50% of the amino acid sequence (Herradon et al., 2019). They consist of two TSR domains with a hinge connection. Each domain consists of three antiparallel β-strands to form β-sheets. The C-terminal domain (CTD) of PTN is the main CS-binding domain, which has an affinity far greater than that of the N-terminal domain (NTD) (Ryan et al., 2016). CTD has two basic residue clusters (cluster 1: K^69^, K^91^, K^92^ and cluster 2: K^84^, K^86^, K^107^). The electrostatic potential diagram showed that the two sides of the β-sheet can be coplanar. According to the PRE data, CS-A preferred cluster 2, while CS-E preferred cluster 1. The data showed that K^54^ in NTD was close to the paramagnetic center, but NTD had only a few residues with side chains and HN atom transfer perturbation. The hydrophobic hinge can arrange two lysines (K^60^ and K^61^) near CTD cluster 1 to participate in the binding of CS. Although there was no clear reason to prove the effect of the C-terminus on the binding of CS to PTN, the affinity of CS-A, but not CS-E, to the C-terminal truncated PTN was greatly reduced. CS-E had a greater affinity than CS-A, which might be the reason why the PTN/MK family was associated with many tumorous inflammations (Weckbach et al., 2018). Unlike PTN, according to STD data, CS-E can simultaneously bind to the two domains of the midkine (Solera et al., 2016).

Tumor necrosis factor-stimulated gene-6 (TSG-6) is a classic HA-binding protein that shows different binding modes with CS compared to HA (Park et al., 2016). The combination of CS and Link- TSG-6 had at least two binding sites, and 4-O-sulfation was preferred. The slow exchange site was similar to the HA-binding site, but there were still some differences due to the sulfation pattern of CS. STD data indicated that there was a second group of rapid exchange binding sites, which were close to the heparin-binding site according to the model based on PRE data. The change in the relaxation rate ratio R2/R1 indicated that the initial combination of CS and Link- TSG-6 can induce dimerization. The dimerization interface and the CS binding site were located on opposite sides, so CS plays a neutralizing role rather than functioning as a bridge in inducing dimerization.

## Dermatan Sulfate

Although DS was similar in structure to CS, the existence of IdoA gave it unparalleled structural flexibility. For example, in combination with hepatocyte growth factor/scattering factor (HGF/SF), the presence or absence of IdoA was the key to the combination of GAG with HGF/SF (Deakin et al., 2009). The binding mode of DS and NK1 (HGF/SF heparin-binding domain) was similar to that of heparin, although the affinity was slightly lower. The binding was concentrated in the N domain. Although crystallographic data proved that the K1 domain was involved in binding, this binding was based on the premise of dimerization. However, the NMR data showed that in solution, the low-molecular-weight GAGs would not induce its dimerization.

Sepuru used medium-length GAG to study the interaction with CXCL1 or CXCL5 in the presence of monomers and dimers through CSP experiments (Sepuru and Rajarathnam, 2019). The two binding sites in CXCL1 with HS were on the opposite sides of the protein, the α-domain (H^19^, K^21^, K^45^, K^60^, K^61^, K^65^) and the β-domain (R^8^, K^29^, R^48^, K^49^). The results showed that CXCL1 and HS were combined in a ratio of 1:2, and ITC experiments verified this result. The binding sites of CXCL1 with CS and DS are located in the γ-domain (R^8^, H^19^, K^21^, K^45^, K^49^). The binding domain of CXCL5 with GAG was similar to that of CXCL1, but there was no obvious specificity for GAG species. Neither CXCL1 nor CXCL5 bound to GAG involved helices, which was different from the previous proposal that helices are an important binding site for the interaction of chemokines that activate CXCR2 with GAG. In the HADDOCK model, the interaction between DS and CXCL1 involved two sulfate groups, two carboxyl groups and two N-acetyl groups, and the interaction model with CXCL5 involved two sulfate groups, one N-acetyl and one hydroxyl group. The molecular docking models of CS and DS with different structures were quite different. They involved different residue-binding groups and positions. This was consistent with the differences in the interaction morphology of GAG with different structures proposed previously. This was also reflected in the combination of CXCL14 and DS (Penk et al., 2019). The binding of DS and heparin with CXCL14 occurred in the C-terminal helix, part of the N-terminus and the transition between the second and third β-sheets (Y^44^-Q^47^). However, the maximum perturbation in the combination of DS and CXCL14 was associated with R^72^, while I^36^ and T^37^ were more affected in terms of heparin. DS and CS also had significant differences in N-terminal disturbances. The interaction between DS and protein was also dependent on chain length and sulfation pattern. In the study of the interaction between tau protein and DS, tau was favored for 6-O-sulfation (Zhao et al., 2017). Disulfated DS had a higher affinity than monosulfated DS, although the affinity of both was less than that of heparin.

Decorin binding protein B (DBPB) bound to DS in a different binding mode than DBPA, mainly through the linker between helices 1 and 2, the C-terminal tail, and the alkaline patch (Feng and Wang, 2015). In the PRE experiment, there were no clear data indicating that the C-terminal tail was involved in binding. It was speculated that this was because the binding occurs at the nonreducing end of DS, while the TEMPO label was at the reducing end of DS. The mutation data showed that the three sites all had a promoting effect on binding, and the C-terminus played a key role in binding. The most obvious difference between DBPB and DBPA was only the C-terminal disulfide bond, which again emphasized the influence of protein structure on binding. Due to the lack of disulfide bonds, the C-terminus could exist in multiple conformations when combined with DS, which was also thermodynamically favorable. Although the BXBB sequence in DBPA remained highly dynamic in DBPB, it did not contribute much to the binding due to the exposure of the C-terminus and the position of the linker in DBPB.

## Hyaluronic Acid

Hyaluronic acid has a different synthesis site (plasma membrane) and a different synthesis form (non-glycoprotein) compared to other GAGs. HA will not undergo further modification; thus, the interaction between it and the protein seems to be structurally specific. The hydrogen bonds and intramolecular hydrogen bonds with water molecules gave it a complex β-sheet structure (Taweechat et al., 2020). In the double helix structure of HA, every two monosccharide flip 180°. HA, as a structural scaffold, widely exists in the epithelial tissue, connective tissue and nerve tissue of vertebrates and regulates the physical and chemical processes of tissue hydration and penetration. The interaction between HA and HA-binding protein (hyaluroadhesin) mediates various physiological activities, such as cell signal transduction, wound repair, tissue regeneration, leukocyte rolling adhesion and inflammation (Fallacara et al., 2018). Most HA-binding proteins belong to the link protein superfamily. Some other proteins (such as receptor for hyaluronan-mediated motility, RHAMM) and peptides (thymosin α1, Tα1) bound to HA are independent of the link module (Naor, 2016).

**FIGURE 5:**
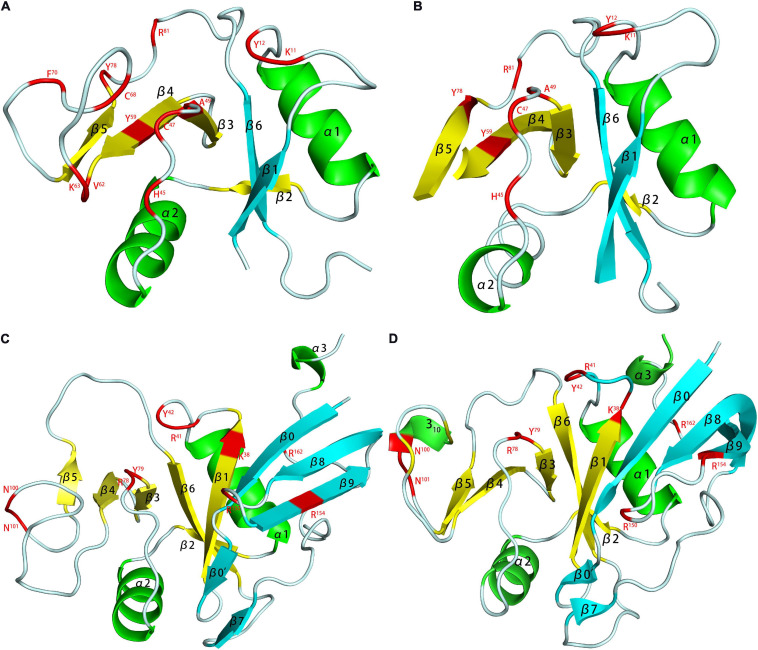
HA binding domains (HABD) of TSG-6 [**(A)** PDB code 1O7B; **(B)** PDB code 2PF5] and CD44 [**(C)** PDB code 1POZ; **(D)** PDB code 1UUH]. In the models, the TSG-6 or CD44 residues participate in binging are shown in red. The HABD of TSG-6 was the only Link module. The link module was structured by two β-sheets and two α-helices. The two β-sheets were composed of four and two β-strands. CD44 extended the β-sheet at the C- and N-termini on the basis of TSG6 (adding four β strands), and the HABD of CD44 was redefined. Unlike the NMR model **(C)**, due to the low charge density caused by the conformational balance, the crystal **(D)** does not have a secondary structure in residues 62-73.

The 14 human link proteins can be divided into three categories (A, B, C) according to their structural composition (Kohda et al., 1996). TSG-6 was the most typical type A Link protein, and its HA-binding domain (HABD) was the only Link module (Figure 5; Day and Milner, 2019). The link module was composed of 100 amino acids and structured by two β-sheets and two α-helices, which were stabilized by two extremely conserved disulfide bonds. The two β-sheets were composed of four and two β-strands. Type B Link protein used CD44 as a template. It extended the β-sheet at the C- and N-termini on the basis of type A (adding four β strands), and the HABD of type B was redefined (Senbanjo and Chellaiah, 2017). The type C link protein was composed of two links in series, both of which participate in binding with HA. This subcategory included aggrecan, versican and HAPLN1-4, but detailed research on its structure is lacking. The binding of HA and protein had very strict requirements on the tertiary structure of the protein. This was most obvious in the type C Link protein, which did not interact with GAGs other than HA. In one study, three link modules were connected in series, but the binding activity with HA was completely lost (Cai et al., 2004).

Kahmann proposed that the binding of Link-TSG-6 and HA was concentrated in the β4/β5 loop. The association was accompanied by the rearrangement of C^47^ and C^68^ disulfide bonds (Kahmann et al., 2000). In the previously proposed B(X)_7_B rule motif (R^5^EARSGKYK^13^), R^5^ and K^13^ had no obvious evidence of involvement in binding, but K^11^ was the main binding residue. In Blundell’s subsequent research, it was shown that the folding of the link module remains unchanged during the combination (Blundell et al., 2003). The largest structural change was found in β4/β5. K^11^ also changed its orientation and became more oriented. For Y^59^ and Y^58^, the benzene rings did not rotate due to ring stacking. Due to the derived polarity of the binding, the two ends of the binding were located at K^11^ and R^81^. Higman proposed that in the free state, the β4/β5 loop of TSG-6 was highly dynamic. In this state, there was a conformation that exposes aromatic residues and captured HA by stacking interactions and then rearranged structural elements, such as the β4/β5 loop (Higman et al., 2007). There were two structural elements that were obviously solidified, one of which was G^10^ located at the corner of α1/β1, and the other was K^54^ of β3/β4. K^54^ was far from the HA-binding site but played an important role in the binding of heparin to TSG-6. Its solidification explained the problem that HA and heparin could not bind to TSG-6 at the same time, although they have different binding sites.

In the 2014 study, HA and hybrid HA of different lengths were used to study the interaction with Link-TSG-6 (Higman et al., 2014). Although the heptasaccharide with the reducing end of GlcA (HA_7_^AA^) had a complete binding structure, the entropy was unfavorable. Therefore, the octasaccharide with the reducing end of GlcNAc (HA_8_^AN^) was defined as the minimum unit required for binding. HSQC data clearly showed that HA_8_^NA^ and HA_7_^AA^ had two binding modes, with the reducing end GlcA bound to K^63^/H^45^ as the dominant one. The affinity of HA_8_^NA^ was twice that of HA_8_^AN^, while the affinity of the two heptasaccharides had no such difference. The reason for the difference in specific affinity is unknown. In the binding model of HA_8_^AN^ and TSG-6, H^45^ and K^63^ appear to be new binding residues. They bound to the reducing terminal disaccharide of the octasaccharide to make the binding tighter. The binding of HA and Link-TSG-6 was mainly through ionic interactions, ring-stacking interactions, hydrogen bonding, van der Waals forces and hydrophobic repulsion. Since the binding occurred on two interfaces, this imposed an inevitable requirement for the distortion of the two glycosidic bonds between the fifth and seventh residues. For heptasaccharides, the significant reduction in the affinity of hexasaccharides might be due to the lack of multiple groups of binding, resulting in instability of the distortion of glycosidic bonds. The CS part of hybrid HA will also be distorted during binding, but due to the lack of structural elements and the lack of hydrogen bonds during binding, the affinity was far lower than that of HA. However, due to the existence of binding, this provided a certain explanation for the chondroprotective function of TSG-6. CS, Heparin and HA had different binding modes with TSG-6, giving TSG-6 complex biological functions.

**FIGURE 6:**
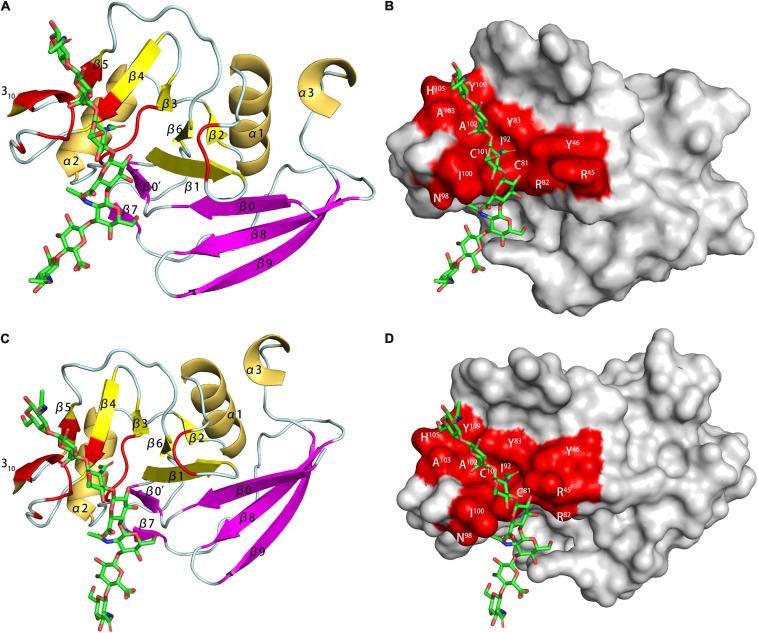
The HA-binding site in mouse CD44. [**(A)** PDB code 2JCQ; **(C)** PDB code 2JCR] The ribbon diagram of mouse CD44 (type A and B complex). **(B,D)** Surface representation of the HA binding site in the type A and B crystal complex.

The HABD in CD44 was mainly located in the link module, C-terminal extension and α1-helix. Two N-linked glycosylation sites (N^25^ and N^100^) were also located in the HABD (Takeda et al., 2003). Teriete pointed out that octasaccharide might be the smallest unit that satisfies all binding requirements (Teriete et al., 2004). All binding sites were located on the same plane, but due to the scattered distribution, there might be two incompatible binding modes. One used N^100^/N^101^ to R^150^/R^154^, similar to the combination of TSG-6 and HA. The other used K^38^/R^162^ as the terminal binding, and the binding was farther away from the charged area. The data showed that the binding is accompanied by a structural rearrangement. Takeda proposed that the parallel sheets of β8 and β0 involved rearrangement, which might be related to the special structure of β8 (Takeda et al., 2006). More thorough structural changes were located at the C-terminal extensions of α3 and β9, and their structure changed from a regular to a randomized structure after the combination. This result was in conflict with crystal studies, which showed that binding did not involve changes in C-terminal extension (Banerji et al., 2007). But unlike other studies, the protein used by Banerji is of mouse origin. And in the model established in this study, the complex is in two conformational equilibrium (type A and B, Figure 6). The difference between the two conformations is the orientation of R^45^ (human CD44 R^41^). Ogino also proposed that CD44 was in the balance of two conformations in the unbound or bound state (Ogino et al., 2010). In the unbound state, it had a regular structure and low HA affinity, which was conducive to cell rolling. In the combined state, it was mainly a random structure with high HA affinity, which was conducive to cell adhesion. The balance of these two states was conducive to the physiological activity of CD44-mediated cell rolling.

In terms of RHAMM, two amino acid clusters were mainly involved in binding with HA: the first was the proposed BX_7_B structure (K^531^-K^541^), and the second was K^553^-K^562^ (Ziebell and Prestwich, 2004). Studies have shown that the second binding site plays a major role in binding. Studies on Tα1 indicated that the binding is mainly related to its terminal L^16^KEKK^20^ (Mandaliti et al., 2017). The combination of HA and these two substances occurred mainly through electrostatic forces, which was different from the role of HA with TSG-6 and CD44. The combination of HA and CD44 was mainly through hydrogen bonding and van der Waals forces, while the combination with TSG-6 was mainly through electrostatic forces and aromatic accumulation.

## Kertan Sulfate

Kertan sulfate is the only GAG without any acidic uronic acid residue, and its interaction with proteins mainly depends on structural characteristics and sulfation modification. KS is mainly distributed in the cornea and cartilage tissue and is divided into three categories (I-III) according to the distribution and connection with glycoproteins (Caterson and Melrose, 2018). KS plays an important role in brain development, neurodevelopment and regeneration, implantation and fertilization and maintains the balance of tissue hydration properties (Ota et al., 2018; Melrose, 2019; Miller et al., 2020). KS has many protein partners, including tyrosine protein kinases, inflammatory cytokines, growth factors, chemokines, cytoskeletal cells, and lectins. Only a few studies of the interaction between KS and protein have been investigated using NMR (Huckerby, 2002).

Galectin 3 (Gal-3) seems to be one of KS’s most tacit partners, and its distribution is extremely close to that of KS. The interaction between full-length Gal-3 and KS has been studied using HSQC; the disturbance was found to be in the β1, β3, β4, β5, β6, and β10 strands, and the β10 strand was the most important strand. The binding domain can be on the S- and F-faces in Gal-3. When the N-terminal tail of Gal-3 was truncated, KS interaction on the S-face became more obvious. The presence of other negatively charged regions did not affect the binding between KS and the Gal-3 S-face according to MD data. In the binding state, the conformations of the F-face and the N-terminal tail were changed. The binding was mainly concentrated on the left side of the S-face, which facilitated its combination with other proteins or heteropolymerization with other galectins. However, the pulse field gradient NMR data showed that KS did not induce oligomerization of Gal-3. Desulfated KS had far less affinity than KS, and the chemical shift disturbances on the F-face and N-terminal tail were greatly reduced.

## Conclusion

Glycosaminoglycans, as common glycoproteins in biological systems, are involved in many physiological and pathological processes. The study of their structure and interaction with proteins has received extensive attention, but the study of molecular perspectives is only the tip of the iceberg. This not only is due to the delay of carbohydrate research but is also related to the limitations of technology. The information produced by NMR is incomparable to all other technologies. For example, it can provide information about the binding affinity constant, on/off chemical exchange rate, binding site and atomic information, but high-precision research is more demanding for technology. In particular, regarding the special existence of GAG, its highly complex structure not only endows it with rich biological functions but also brings incomparable difficulties for research. The study of the interactions between GAG and proteins using NMR is based on complete structural characterizations of GAG and/or proteins, which face huge obstacles. Biosynthesis carriers of GAG are difficult to find, while chemical and enzymatic syntheses are limited to a few scientists. This in turn makes it difficult to obtain isotope-labeled GAG. Because the binding of GAG and protein has obvious multibinding characteristics, it will cause oligomerization and even precipitation. The application of NMR technology is mainly limited by several factors, including the length of the oligosaccharides, the molecular weight of the proteins, and the concentration range and stability of the complex. However, with the renewal and iteration of technology, the rise of high magnetic flux nuclear magnetic spectrometry and enzymatic chemical synthesis has injected a steady stream of vitality into interaction research. The study of the interaction between GAG and proteins is helpful for understanding various physiological and pathological mechanisms and has a huge impetus for drug development.

## Author Contributions

CB and LJ participated in preparation, creation, initial draft writing and review of this article. Both authors contributed to the article and approved the submitted version.

## Conflict of Interest

The authors declare that the research was conducted in the absence of any commercial or financial relationships that could be construed as a potential conflict of interest.
